# Filtration mapping as complete Bell state analyzer for bosonic particles

**DOI:** 10.1038/s41598-021-93679-7

**Published:** 2021-07-09

**Authors:** A. V. Kozubov, A. A. Gaidash, A. D. Kiselev, G. P. Miroshnichenko

**Affiliations:** 1 Department of Mathematical Methods for Quantum Technologies, Steklov Mathematical Institute of Russian Academy of Sciences, 119991 Moscow, Russia; 2 Laboratory of Quantum Processes and Measurements, ITMO University, Kadetskaya Line 3b, 199034 Saint Petersburg, Russia; 3 Faculty of Physics, St. Petersburg State University, 199034 Saint Petersburg, Russia; 4 Faculty of Laser Photonics and Optoelectronics, ITMO University, 49 Kronverksky Pr., 197101 Saint Petersburg, Russia

**Keywords:** Quantum physics, Quantum information, Qubits, Theoretical physics

## Abstract

In this paper, we present the approach to complete Bell state analysis based on filtering mapping. The key distinctive feature of this appoach is that it avoids complications related to using either hyperentanglement or representation of the Bell states as concatenated Greenber–Horne–Zeilinger (C-GHZ) state to perform discrimination procedure. We describe two techniques developed within the suggested approach and based on two-step algorithms with two different types of filtration mapping which can be called the non-demolition and semi-demolition filtrations. In the method involving non-demolition filtration measurement the filtration process employs cross-Kerr nonlinearity and the probe mode to distinguish between the two pairs of the Bell states. In the case of semi-demolition measurement, the two states are unambiguously discriminated and hence destroyed, whereas filtraton keeps the other two states intact. We show that the measurement that destroys the single photon subspace in every mode and preserves the superposition of zero and two photons can be realized with discrete photodetection based on microresonator with atoms.

## Introduction

Entanglement is one of the most curious and exciting phenomena in the quantum world. Its applications can be found in different areas of the quantum information theory. It has been widely used in a variety of related fields such as the theory of quantum channels, quantum key distribution (QKD)^1,2^, quantum teleportation^3^, dense coding^4^, quantum secure direct communication^5–12^, distributed secure quantum machine learning^13^, quantum repeaters^14–16^ and quantum computation^17–19^. The well-known Bell states being a conceptually important simple example of maximally entangled two-qubit states play a crucial role in understanding of entanglement as a valuable resource that may be utilized to perform novel information processing tasks.

In this paper we deal with the problem of unambiguous identification of the Bell states which is one of the central problems concerning the Bell states. In particular, the related procedure of unambiguous identification is among the key factors that determine the performance of such protocols as measurement-device-independent QKD, quantum teleportation and dense coding. An important point is that all these protocols are mostly based on photonic qubits.

It is well known that in the case of photonic systems one cannot distinguish all the four Bell states unambiguously using only linear optical elements and detectors^20,21^. The reason is that photons obey the Bose–Einstein statistics. Thus efficiency of the above listed protocols appears to be crucially reduced. The most common Bell states analyzer for the photonic states was proposed in^22^ and is known as the Innsbruck scheme. As is estimated in^23^, for this type of the Bell states, it is impossible to get more than 50% success rate using tools of linear optics.

The auxiliary-photon-enhanced scheme suggested in^24^ to improve the probability of discrimination uses a pair of ancillary entangled photons to increase the probability up to 75%. More generally, according to^25^, introducing $2^N-2$ ancillary photons may enhance the success rate of linear-optic measurements up to $1-1/2^N$.

Bell state analysis has also been the subject of intense studies as part of quantum information processing assisted by nonlinear interactions^26^. In^27^, the process of optical sum frequency generation was utilized to convert two-photon polarization Bell states into single-photon states and unambiguously detect them. Though this technique is quite elegant, it still leads to a rather inefficient protocol. Nondestructive discrimination between the singlet state and the triplet Bell states after their conversion on a beam splitter was demonstrated in^28^ using the nonlinear cross-Kerr effect. Typically, in real crystals, nonlinear interactions are weak and one of the common difficulties faced by applications of the methods based on optical nonlinear processes is their poor efficiency. The method to get around this problem suggested in^29^ is based on electromagnetically induced transparency that yields giant resonantly enhanced Kerr nonlinearity.

The optical Kerr effect has also been used in devices performing quantum nondemolition (QND) measurements^30,31^. QND detectors using cross-Kerr nonlinearity lie at the heart of the QND method employed in^32^ to construct a nearly deterministic controlled-CNOT gate. In^28^, this method was used for the Bell-state analysis. It was extended to the cases of Greenberger–Horne–Zeiglinger (GHZ) state and hyperentangled Bell state analyzers in^33,34^, respectively. Similar to^32,33^, the method for complete polarization logic Bell-state analysis developed in^35^ uses the parity-check QND measurements constructed by the cross-Kerr nonlinearity. Another possible way to realize QND measurement and utilize it for Bell state analyzer was proposed in^36^.

The approach using the Bell states embedded in a larger Hilbert that represent the so-called hyperentangled states which are entangled in more than one degree of freedom^37^ allows to distinguish the four Bell states by means of linear optic elements^38^. For instance, the experimental setup of^39^, in addition to the polarization degree of freedom, employed intrinsic time–energy correlations of the down-converted pairs of photons. Alternatively, the photons may additionally be entangled in the wave vector (linear momentum) giving the direction of propagation from a nonlinear crystal during spontaneous parametric down-conversion^40–42^. Nonlinear optical properties of a quantum dot-cavity system was utilized for generation of hyperentangled spatially polarized photons in^43^. The dense-coding experiment where the channel capacity was greatly improved using the two-photon states that are simultaneously entangled in spin and orbital angular momentum was reported in^44^.

Various methods for complete analysis of hyperentangled Bell states assisted by nonlinear interactions and auxiliary entanglement were reported in recent studies^45–48^. Hyperentanglement-assisted versions of the GHZ state analysis are developed in^49–55^.

In this paper we show how to construct the bosonic Bell state analyzer using the approach based on filtration mapping. Our goal is to explore the possibility of complete discrimination the four two-photon polarization Bell states without recourse to additional degrees of freedom. The latter is the distinguishing feature of our protocol as compared to a variety of the above discussed hyperentanglement protocols^34,39–42,45–48^. We also describe two different types of filtration leading to different realizations of the two-step procedure for completely deterministic Bell state measurement of the polarization qubits.

## Results

### Filtration mapping

It is well-known that Bell states form the orthogonal basis. Thus these states should be distinguished unambiguously and the mathematical representation of the corresponding measurement poses no problem. From the above discussion, owing to the bosonic nature of the photonic Bell states, complete analysis of such states requires methods that go beyond the scope of linear optics such as approaches using either non-linear elements or additional degrees of freedom.

In this section, we shall present the new approach that provides a two-step algorithm capable of discriminating all four bosonic Bell states without introducing additional degrees of freedom. Mathematical structure underlying the two-step procedure can be illustrated in the simple and elegant way using the polar decomposition of positive semi-definite operators. It can be described as a completely-positive trace-preserving (CPTP) map determined by a family of positive semi-definite operators $\{A_x\}_{x\in {\mathscr {X}}}$:

1 $$\begin{aligned} {\mathbb {I}}=\sum _{x\in {\mathscr {X}}} A_x. \end{aligned}$$

The corresponding Kraus operators should meet the condition $K_x^{\dagger }K_x=A_x$ and are not uniquely defined. The polar decomposition for these operators

2 $$\begin{aligned} K_x= U^x \sqrt{A_x}, \end{aligned}$$

involves a unitary operator $U^x$ that can be chosen at will.

In our case, the amplitude part of $$K_x$$, $$\sqrt{A_x}$$, represents the filtration process that extracts the classical bit of information. The kernel of the filtration operators is spanned by the pair of Bell states which are not allowed to pass the filter. The unitary $$ U^x$$ in its turn provides additional unitary transformation needed to discriminate between the states inside the pair passed the filter. In what follows we describe two different techniques that can be viewed as an implementation of the general two-step method.

### Bell state analyzer with non-demolition filtration

A probe light field prepared in the coherent state is known to acquire the phase shift induced by the cross-Kerr nonlinear interaction^26,28,32^. In our scheme this nonlinear phase shift underlies the parity-check QND measurements used for filtration of the Bell states.

In previous studies^34,46,48^ such measurements were employed for complete analysis of hyperentangled Bell states. By contrast, our method does not rely on hyperentaglement. Instead of using QND measurement for exact identification of the Bell states, we perform it only during the filtration process to separate two pairs from each other according to the parity information obtained from homodyne detection of the probe (auxiliary) beam prepared in the coherent state. The filtration idea is similar to one proposed in^36^. However, the approach proposed in^36^ utilizes the QND measurement (based on the Faraday effect) that differ from the one in the paper. The method suggested in^36^ requires tuning the signal photon into precise resonance with the quantum dot levels. By contrast, the Kerr effect utilized in our approach is not resonant and can be applied to Bell state photons with different frequencies. After separating two pairs of the Bell states from each other, the filtration procedure leaves the passed states intact and yields the classical bit of information. This bit defines the unitary operation subsequently applied at the second step to accomplish discrimination task. In a sense such approach bears some resemblance to the protocol used for teleportation of quantum states.

Thus our method involves the two following steps:

1. *Filtration step.* The states are only allocated pairs from each other providing the classical bit of information.
2. *Unitary transformation.* According to the classical bit obtained from the filtration we choose the appropriate unitary transformation which allows to discriminate between states inside the pair.

#### State preparation

The two-photon polarization Bell states to be distinguished can be written in the following form:

3 $$\begin{aligned} |\Psi ^\pm \rangle= & {} \frac{1}{\sqrt{2}}(a_{H_1}^\dagger a_{V_2}^\dagger \pm a_{V_1}^\dagger a_{H_2}^\dagger ) |0\rangle ,\end{aligned}$$

4 $$\begin{aligned} |\Phi ^\pm \rangle= & {} \frac{1}{\sqrt{2}}(a_{H_1}^\dagger a_{H_2}^\dagger \pm a_{V_1}^\dagger a_{V_2}^\dagger ) |0\rangle , \end{aligned}$$

where $a_{H_1}^\dagger , a_{V_1}^\dagger$ and $a_{H_2}^\dagger , a_{V_2}^\dagger$ are the creation operators for the horizontal and vertical modes of the first and the second spatial modes, respectively; $|0\rangle$ is the vacuum state. In order to successfully distinguish between the four states it would be convenient to introduce spatial (rail) coding notation and express the latter states in it; this transformation can be done using two separate polarization beam splitters located in each of the two initial spatial modes. We apply corresponding unitary transformations at the output of the polarization beam splitters (by a set of wave plates) so that photons at the four output spatial modes (rails) have the same polarization. Thus we obtain the four spatial modes labelled by the integers ranged from 1 to 4:

5 $$\begin{aligned} a_{H1}^\dagger =a_1^\dagger ,\quad a_{V1}^\dagger =a_2^\dagger ,\quad a_{H2}^\dagger =a_3^\dagger ,\quad a_{V2}^\dagger =a_4^\dagger . \end{aligned}$$

By using the rail notations for the Fock states

6 $$\begin{aligned} a_{i}^\dagger |0\rangle \equiv |1_i\rangle ,\quad a_{i}^\dagger a_{j}^\dagger |0\rangle \equiv |1_{ij}\rangle ,\quad (a_{i}^\dagger )^2 |0\rangle \equiv |2_{i}\rangle , \end{aligned}$$

the Bell states can be conveniently rewritten in the following form:

7 $$\begin{aligned} |\Psi ^{+}\rangle= & {} |B_1\rangle =\frac{1}{\sqrt{2}}(a_{1}^\dagger a_{4}^\dagger + a_{2}^\dagger a_{3}^\dagger ) |0\rangle \equiv \frac{1}{\sqrt{2}}\left( |1_{14}\rangle + |1_{23}\rangle \right) , \end{aligned}$$

8 $$\begin{aligned} |\Psi ^-\rangle= & {} |B_2\rangle =\frac{1}{\sqrt{2}}(a_{1}^\dagger a_{4}^\dagger - a_{2}^\dagger a_{3}^\dagger ) |0\rangle \equiv \frac{1}{\sqrt{2}}\left( |1_{14}\rangle - |1_{23}\rangle \right) ,\end{aligned}$$

9 $$\begin{aligned} |\Phi ^+\rangle= & {} |B_3\rangle =\frac{1}{\sqrt{2}}(a_{1}^\dagger a_{3}^\dagger + a_{2}^\dagger a_{4}^\dagger ) |0\rangle \equiv \frac{1}{\sqrt{2}}\left( |1_{13}\rangle + |1_{24}\rangle \right) ,\end{aligned}$$

10 $$\begin{aligned} |\Phi ^-\rangle= & {} |B_4\rangle =\frac{1}{\sqrt{2}}(a_{1}^\dagger a_{3}^\dagger - a_{2}^\dagger a_{4}^\dagger ) |0\rangle \equiv \frac{1}{\sqrt{2}}\left( |1_{13}\rangle - |1_{24}\rangle \right) . \end{aligned}$$

#### Filtration

Let us introduce the filtering operation that discriminates between the two pairs: $\{{\vert }{B_1}{\rangle },{\vert }{B_2}{\rangle }\}\in \mathrm {span}({\vert }{1_{14}}{\rangle },{\vert }{1_{23}}{\rangle })$ and $\{{\vert }{B_3}{\rangle },{\vert }{B_4}{\rangle }\}\in \mathrm {span}({\vert }{1_{13}}{\rangle },{\vert }{1_{24}}{\rangle })$. The relations describing the corresponding filtering operators, ${\hat{F}}_{12}$ and ${\hat{F}}_{34}$, are given by:

11 $$\begin{aligned} {\hat{F}}_{12} |B_1\rangle = |B_1\rangle ,\ {\hat{F}}_{12} |B_2\rangle= & {} |B_2\rangle ,\ {\hat{F}}_{12} |B_3\rangle = {\hat{F}}_{12} |B_4\rangle =0, \end{aligned}$$

12 $$\begin{aligned} {\hat{F}}_{34} |B_3\rangle = |B_3\rangle ,\ {\hat{F}}_{34} |B_4\rangle= & {} |B_4\rangle ,\ {\hat{F}}_{34} |B_1\rangle = {\hat{F}}_{34} |B_2\rangle =0. \end{aligned}$$

Mathematically, these equations imply that linear combinations of the states $|B_3\rangle$ and $|B_4\rangle$ ($|B_1\rangle$ and $|B_2\rangle$) form the kernel of the operator ${\hat{F}}_{12}$ (${\hat{F}}_{34}$):

13 $$\begin{aligned} \mathrm {ker}\, {\hat{F}}_{12}={\text {span}}(|B_3\rangle ,|B_4\rangle ), \quad \mathrm {ker}\, {\hat{F}}_{34}={\text {span}}(|B_1\rangle ,|B_2\rangle ). \end{aligned}$$

Since the kernels of this operators do not overlap, the pairs of the Bell states can be identified unambiguously. In other words, filtering operators ${\hat{F}}_{12}$ and ${\hat{F}}_{34}$ unambiguously identify the pairs of states $|B_1\rangle$ and $|B_2\rangle$, $|B_3\rangle$ and $|B_4\rangle$. Thus,

14 $$\begin{aligned} {\hat{F}}_{12}^\dagger {\hat{F}}_{12}= & {} |B_1\rangle \langle B_1|+|B_2\rangle \langle B_2|, \end{aligned}$$

15 $$\begin{aligned} {\hat{F}}_{34}^\dagger {\hat{F}}_{34}= & {} |B_3\rangle \langle B_3|+|B_4\rangle \langle B_4|. \end{aligned}$$

Filtration operations can be performed by the parity-check measurements using the cross-Kerr nonlinearity. These QND measurements involve the probe (auxiliary) beam prepared in the coherent state ${\vert }{\alpha _p}{\rangle }$ assuming that the Kerr-induced dynamics of the states of the combined signal-probe system, ${\vert }{B_i}{\rangle }\otimes {\vert }{\alpha _p}{\rangle }$, is governed by the unitary (see, e.g.,^26^)

16 $$\begin{aligned} {\hat{U}}_{\mathrm {Kerr}}=\exp \left\{ \sum _{i=1}^4\Theta _i{\hat{n}}_i{\hat{n}}_p\right\} , \end{aligned}$$

where ${\hat{n}}_i=a_{i}^\dagger a_{i}$ and ${\hat{n}}_p=a_{p}^\dagger a_{p}$ is the photon number operator for the probe mode. Without loss of generality we may assume that the presence of photons in the channels 1 and 3 produces identical phase shifts of the coherent state amplitude: $\Theta _1=\Theta _3=\theta /2$, whereas the phase shift for the photons in the channels 2 and 4 is of opposite sign: $\Theta _2=\Theta _4=-\theta /2$. So, we have

17 $$\begin{aligned} {\hat{U}}_{\mathrm {Kerr}}(|B_{1,2}\rangle \otimes |\alpha _p\rangle )= & {} |B_{1,2}\rangle \otimes |\alpha _p\rangle . \end{aligned}$$

18 $$\begin{aligned} {\hat{U}}_{\mathrm {Kerr}}(|B_{3,4}\rangle \otimes |\alpha _p\rangle )= & {} \frac{1}{\sqrt{2}} \left\{ {\vert }{1_{13}}{\rangle }\otimes |\alpha _p\mathrm {e}^{i\theta }\rangle \pm {\vert }{1_{24}}{\rangle }\otimes |\alpha _p\mathrm {e}^{-i\theta }\rangle \right\} . \end{aligned}$$

Homodyne detection can be utilized as the well-known standard method to measure phase sensitive properties of the probe field^56^. In this method the probe field is combined with sufficiently intense reference field known as the local oscillator by a beam splitter and the fields in the two output arms are collected by the two photodetectors. The balanced homodyne scheme represents the case where the measured signal is proportional to the difference of photocurrents registered in the arms after a lossless 50:50 beam splitter. This scheme generally amounts to measurements of the values of the quadrature-component operator ${\hat{x}}_p(\varphi )=a_{p}^\dagger \mathrm {e}^{i\varphi } +a_{p}\mathrm {e}^{-i\varphi }$, where the quadrature phase $\varphi$ is determined by the phase difference between the probe field and the local oscillator.

It can be shown that, for the X quadrature-component operator ${\hat{x}}_p(0)\equiv {\hat{x}}_p=a_{p}^\dagger +a_{p}$ with $\varphi =0$, the projection of the probe field coherent states ${\vert }{\alpha _p\mathrm {e}^{\pm i\theta }}{\rangle }$ on the eigenvector ${\vert }{x_p}{\rangle }$ of ${\hat{x}}_p$ (${\hat{x}}_p{\vert }{x_p}{\rangle }={x}_p{\vert }{x_p}{\rangle }$) is given by^56^:

19 $$\begin{aligned} \langle {x_p|\alpha _p\mathrm {e}^{\pm i\theta }}\rangle =(2\pi )^{-1/4}\mathrm {e}^{-(x_p/2-\alpha _p\cos \theta )^2}\mathrm {e}^{\pm i\Phi }, \quad \Phi =\alpha _p\sin \theta (x_p-\alpha _p\cos \theta ). \end{aligned}$$

Clearly, for the Gaussian probability density distribution $f_G(x_p;\theta )=|\langle {x_p|\alpha _p\mathrm {e}^{\pm i\theta }}\rangle |^2$, the mean value is $\langle {x_p}\rangle (\theta )=2\alpha _p\cos \theta$ and $x_{\mathrm {m}}=\alpha _p(1+\cos \theta )$ is the midpoint of the interval ranged from $\langle {x_p}\rangle (\theta )$ to $\langle {x_p}\rangle (0)$. When $\theta =0$ (the measured value of $x_p$ is above $x_{\mathrm {m}}$), the states of the first pair after the homodyne measurements are filtered to ${\vert }{{\tilde{B}}_{1,2}}{\rangle }={\vert }{B_{1,2}}{\rangle }$, whereas in the opposite case with nonvanishing $\theta$ (the measured value of $x_p$ is below $x_{\mathrm {m}}$) the filtered states of the second pair undergo a unitary transformation

20 $$\begin{aligned} {\vert }{{\tilde{B}}_{3,4}}{\rangle }= {\hat{U}}_{ij}(\Phi ){\vert }{B_{3,4}}{\rangle }, \quad {\hat{U}}_{ij}(\Phi )=\mathrm {e}^{i\Phi ({\hat{n}}_i-{\hat{n}}_j)}, \quad i\in \{1,3\},\, j\in \{2,4\}, \end{aligned}$$

that can be easily compensated provided the phase $\Phi$ is known from the measurements of $x_p$. The probability of filtration errors can be estimated from the midpoint $x_{\mathrm {m}}$ and the tails of the Gaussian distributions $f_G(x;0)$ and $f_G(x;\theta )$ giving $P_{\mathrm {err}}=\mathrm {erfc}(\sqrt{2}\alpha _p\sin ^2(\theta /2))/2$. It turns out that $P_{\mathrm {err}}$ is below $3\times 10^{-5}$ at $\alpha _p\sin ^2(\theta /2)>2$.

Note that the idea of parity-check measurement is widely used in different areas of quantum information theory. For example, similar technique was implemented for non-local Bell state measurement^26,57^. Moreover, introduced in this paper consideration of filtering operation is similar to the Fock filtering introduced in^58^. However, an important point is that, in contrast to^58^, we do not use the nonlinearity for identification of exact Bell state. In our scheme it is just the preparatory step before the final discrimination.

**Figure 1:**
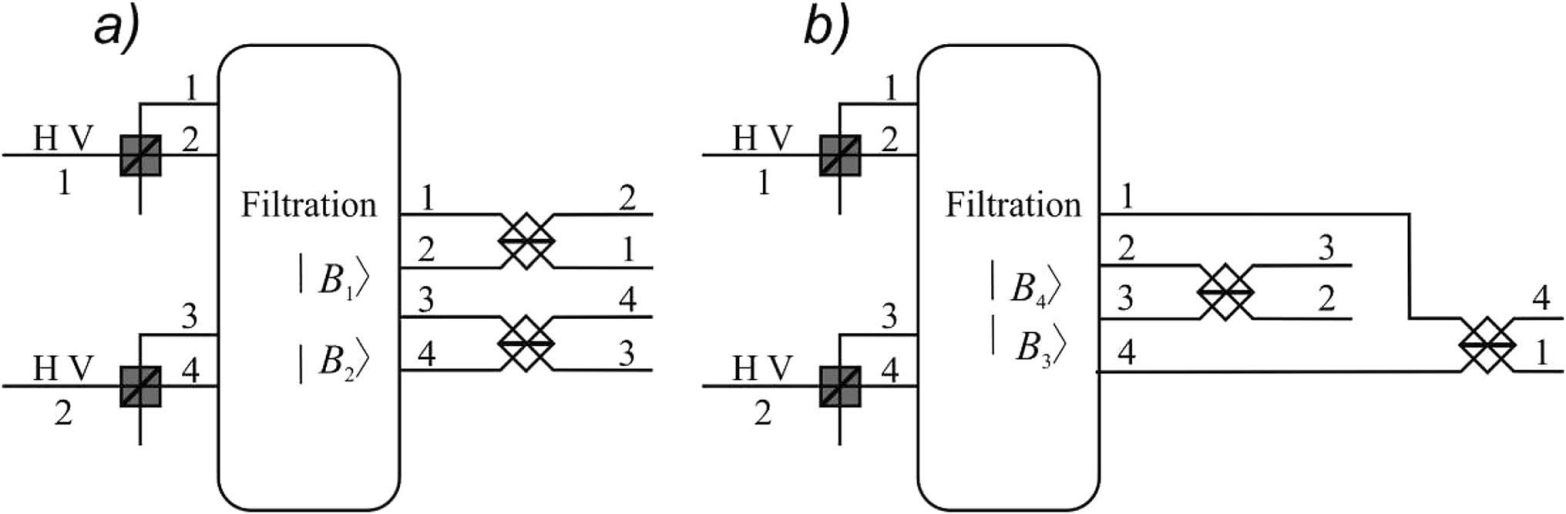
Principle scheme for Bell state measurement with filtration operation based on non-demolition filtration. With grey and white colours polarization and 50:50 beam splitters are denoted respectively. The filtering operation is described with Eqs. (11)–(12), respectively. The unitary transformations for the states $|B_1\rangle , |B_2\rangle$ (**a**) and $|B_3\rangle , |B_4\rangle$ (**b**) according to the classical bit of information are presented.

#### Unitary transformation

After filtration, our task performed at the second step is to discriminate between the states of the filtrated pairs. Let us begin with the discrimination between the Bell states of the first pair $\{{\vert }{B_1}{\rangle },{\vert }{B_2}{\rangle }\}$. Referring to Fig. 1 (left), there are two 50:50 beam splitters connecting the channels 1 and 2, 3 and 4, respectively. From the well-known relations between input and output creation operators for the beam splitters

21 $$\begin{aligned} \begin{pmatrix} {a}^{\dagger }_1\\ {a}^{\dagger }_2 \end{pmatrix}\mapsto \begin{pmatrix} {b}^{\dagger }_1\\ {b}^{\dagger }_2 \end{pmatrix} = \mathbf {C}\begin{pmatrix} {a}^{\dagger }_1\\ {a}^{\dagger }_2 \end{pmatrix}, \quad \begin{pmatrix} {a}^{\dagger }_3\\ {a}^{\dagger }_4 \end{pmatrix}\mapsto \begin{pmatrix} {b}^{\dagger }_3\\ {b}^{\dagger }_4 \end{pmatrix} = \mathbf {C}\begin{pmatrix} {a}^{\dagger }_3\\ {a}^{\dagger }_4 \end{pmatrix}, \quad \mathbf {C}=\frac{1}{\sqrt{2}} \begin{pmatrix} 1&{} -1\\ 1&{}1 \end{pmatrix} \end{aligned}$$

the states $|B_1\rangle$ and $|B_2\rangle$ transform as follows:

22 $$\begin{aligned} {\vert }{B_1}{\rangle }\mapsto \frac{1}{\sqrt{2}} ({b}^{\dagger }_2{b}^{\dagger }_4-{b}^{\dagger }_1{b}^{\dagger }_3){\vert }{0}{\rangle }= \frac{1}{\sqrt{2}}({\vert }{1_{24}}{\rangle }-{\vert }{1_{13}}{\rangle }), \quad {\vert }{B_2}{\rangle }\mapsto \frac{1}{\sqrt{2}} ({b}^{\dagger }_1{b}^{\dagger }_4+{b}^{\dagger }_2{b}^{\dagger }_3){\vert }{0}{\rangle }= \frac{1}{\sqrt{2}}({\vert }{1_{14}}{\rangle }+{\vert }{1_{23}}{\rangle }). \end{aligned}$$

Similarly, for the second pair, in order to discriminate the states $|B_3\rangle$ and $|B_4\rangle$ we can introduce the beam splitters connecting the channels 1 and 4, 2 and 3, respectively (see Fig. 1 (right)). The input–output relations

23 $$\begin{aligned} \begin{pmatrix} {a}^{\dagger }_1\\ {a}^{\dagger }_4 \end{pmatrix}\mapsto \begin{pmatrix} {b}^{\dagger }_1\\ {b}^{\dagger }_4 \end{pmatrix} = \mathbf {C}\begin{pmatrix} {a}^{\dagger }_1\\ {a}^{\dagger }_4 \end{pmatrix}, \quad \begin{pmatrix} {a}^{\dagger }_3\\ {a}^{\dagger }_2 \end{pmatrix}\mapsto \begin{pmatrix} {b}^{\dagger }_3\\ {b}^{\dagger }_2 \end{pmatrix} = \mathbf {C}\begin{pmatrix} {a}^{\dagger }_3\\ {a}^{\dagger }_2 \end{pmatrix}, \end{aligned}$$

can now be used to obtain the transformed states

24 $$\begin{aligned} {\vert }{B_3}{\rangle }\mapsto \frac{1}{\sqrt{2}}({\vert }{1_{34}}{\rangle }-{\vert }{1_{12}}{\rangle }), \quad {\vert }{B_4}{\rangle }\mapsto \frac{1}{\sqrt{2}}({\vert }{1_{13}}{\rangle }-{\vert }{1_{24}}{\rangle }), \end{aligned}$$

The states (22) and (24) now have different photon distributions between modes and thus can be unambiguously discriminated using standard quadratic photodetectors. The important thing about it is that we still have four channels in our model and we only change the unitary operator.

### Bell state analyzer with semi-demolition measurement

An alternative method of discrimination can be described using the well-known Innsbruck scheme for Bell state measurement^22^ and semi-destructive measurement. Though this approach bears some resemblance to the one described in the previous sections, it is based on the different type of filtration. In brief the algorithm is as follows:

1. *Filtering step.* This filtering operation is quite different compared to the one above. It also provides the classical bit of information: classical bit “0” define the states $|B_1\rangle$ and $|B_2\rangle$, and the classical bit “1” shows the second pair of states. These two classical bits are used to define the additional unitary operations which are required to discriminate between the states in pairs.
2. *Unitary transformation.* In this method, the unitary transformation is applied only if appropriate bit was obtained on the filtration step. When the classical bit is “0”, the states $|B_1\rangle$ and $|B_2\rangle$ are already distinguished and thus destroyed. In the case of classical bit “1” one should apply appropriate unitary operation.

#### State preparation and filtration

According to the Innsbruck scheme of Bell state analyzer the states before first measurement can be described as follows^22^:

25 $$\begin{aligned} |\Psi ^+\rangle \rightarrow |D_1\rangle= & {} \frac{1}{\sqrt{2}}\left( a_{3}^\dagger a_{4}^\dagger - a_{1}^\dagger a_{2}^\dagger \right) |0\rangle , \end{aligned}$$

26 $$\begin{aligned} |\Psi ^-\rangle \rightarrow |D_2\rangle= & {} \frac{1}{\sqrt{2}}\left( a_{1}^\dagger a_{4}^\dagger - a_{3}^\dagger a_{2}^\dagger \right) |0\rangle , \end{aligned}$$

27 $$\begin{aligned} |\Phi ^+\rangle \rightarrow |D_3\rangle= & {} \frac{1}{2\sqrt{2}}\left( a_{3}^{\dagger 2}- a_{1}^{\dagger 2}+ a_{4}^{\dagger 2} -a_{2}^{\dagger 2}\right) |0\rangle , \end{aligned}$$

28 $$\begin{aligned} |\Phi ^-\rangle \rightarrow |D_3\rangle= & {} \frac{1}{2\sqrt{2}}\left( a_{3}^{\dagger 2}- a_{1}^{\dagger 2} - a_{4}^{\dagger 2} +a_{2}^{\dagger 2}\right) |0\rangle . \end{aligned}$$

**Figure 2:**
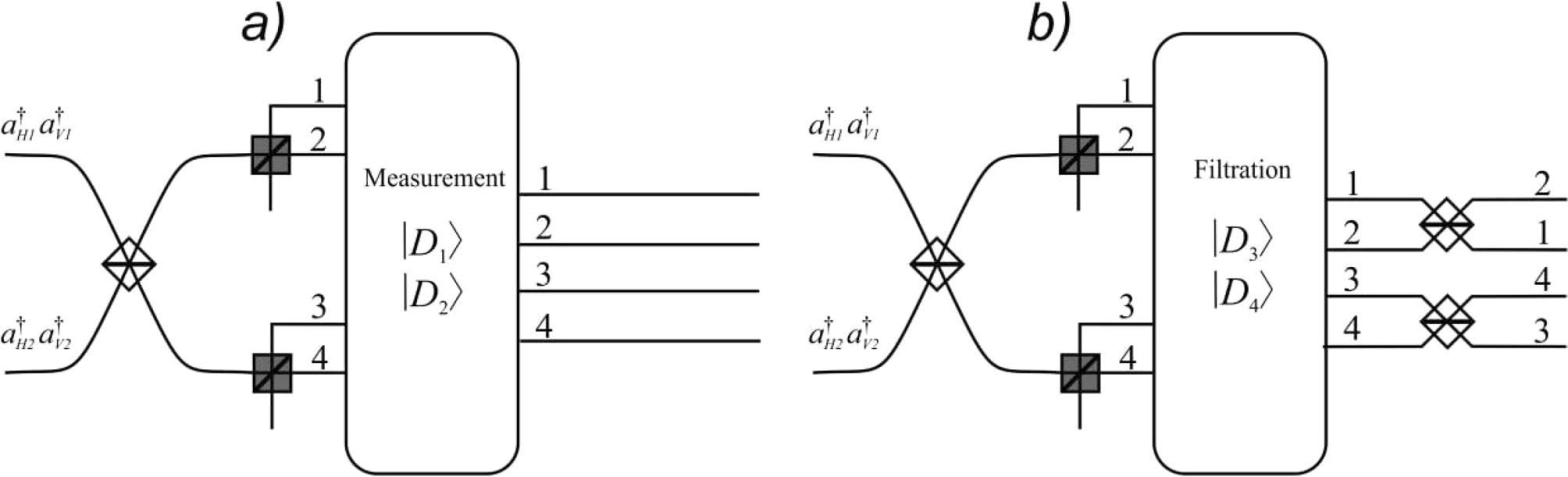
Principle scheme for Bell state measurement with filtration operation based on semi-demolition measurement. With grey and white colours polarization and 50:50 beam splitters are denoted, respectively. The measurement (**a**) and filtering (**b**) operations are described with Eq. (29), respectively.

This transformation can be realized utilizing consistently one 50:50 and two polarization beam splitters as it is shown on Fig. 2

The prime objective of the filtering protocol is to generate the value of the classical bit “0” if the filter has determined a pair of states $|D_1\rangle$ and $|D_2\rangle$, and values “1” for the detected pair of states $|D_3\rangle$ and $|D_4\rangle$. By contrast to the previous protocol, the filter operators are assumed to be of the following form:

29 $$\begin{aligned} F_1=|0\rangle \langle D_1|,\quad F_2=|0\rangle \langle D_2|, \quad F_3=|D_3\rangle \langle D_3|+|D_4\rangle \langle D_4|. \end{aligned}$$

Clearly, in the subspace $\mathrm {span}({\vert }{D_1}{\rangle },{\vert }{D_2}{\rangle },{\vert }{D_3}{\rangle },{\vert }{D_4}{\rangle })$, the completeness condition

30 $$\begin{aligned} {\mathbb {I}}=\sum _{k=1}^3 F_k^\dagger F_k \end{aligned}$$

is satisfied. From Eq. (29), the filtration operators act as:

31 $$\begin{aligned} F_1|D_1\rangle= & {} |0\rangle ,\quad F_2|D_2\rangle =|0\rangle , \end{aligned}$$

32 $$\begin{aligned} F_3|D_3\rangle= & {} |D_3\rangle ,\quad F_3|D_4\rangle =|D_4\rangle \end{aligned}$$

Other states are destroyed under the action of these operators. The operators $$ F_1$$ and $$F_2$$ discriminate the corresponding states $$|D_1\rangle $$ and $$|D_2\rangle $$ by converting them to the vacuum. The states are discriminated between each other according to the configuration of detection clicks. In case when detectors 1 and 2 or 3 and 4 click state $$|D_1\rangle $$ is discriminated. Otherwise, when detectors 1 and 4 or 2 and 3 click state $$|D_2\rangle $$ is discriminated The operator $$F_3$$ discriminates the pair $$|D_3\rangle $$ and $$|D_4\rangle $$ from $$|D_1\rangle $$ and $$|D_1\rangle $$ without state demolition. This event is assigned the value of the classic bit “1”. According to described property we call proposed measurement semi-demolition. The measurement in principle can be performed using discrete photodetection based on microresonator with atoms^59^.

More specifically, the detector represents a microresonator containing a cluster of *N* atoms. The centers of mass of atoms are localized at points ${r}_n$ and are at rest. The microresonator contains a quantum radiation mode and is assumed to be single-mode. The interaction of atoms in the detector with the mode can be described by the Bonifacio model^60^. The primary goal of the detector is to discriminate between one- and two-photon states.

For our purposes, it is sufficient to find the atom-reduced Kraus evolution operators (transformers)^61^ describing the transformation of the reduced density matrix of the mode conditioned by the result of measuring the energy state of the packet atoms. The detectors are adjusted by tuning the interaction time $t=T_{int}$, the number of atoms in a packet *N*, and the interaction parameter $\lambda$. According to^59^, the operators are as follows:

33 $$\begin{aligned} K_0= & {} |0\rangle \langle 0| +\cos (\lambda \sqrt{N}t)|1\rangle \langle 1|\nonumber \\&+\left( \frac{N}{2N-1}(\cos (\lambda \sqrt{4N-2}t)-1)+1\right) |2\rangle \langle 2| \end{aligned}$$

34 $$\begin{aligned} K_1= & {} -i\left( \sin (\lambda \sqrt{N}t)|0\rangle \langle 1|+\sqrt{\frac{N}{2N-1}}\sin (\lambda \sqrt{4n-2}t)|1\rangle \langle 2|\right) \end{aligned}$$

35 $$\begin{aligned} K_2= & {} \frac{\sqrt{N(N-1)}}{4N-2}(\cos (\lambda \sqrt{4N-2}t)-1)|0\rangle \langle 2| \end{aligned}$$

To choose the appropriate construction of the following operators there are two conditions

36 $$\begin{aligned} {\left\{ \begin{array}{ll} \cos (\lambda \sqrt{4N-2}t)=1,\\ \cos (\lambda \sqrt{N}t)=0 \end{array}\right. } \end{aligned}$$

that have to be satisfied. Before performing the instant selective measurement of the states of probe atoms in the packet one should keep the entangled photons in microresonators during the interaction time $t =T_{int}$. Under conditions (36), the detection of an atom in the excited state indicates unambiguously that the one-photon mode state was present in the resonator at the measurement moment. So, the above conditions give the proposed *semi-demolition* measurement.

We can now choose the parameters so as to meet the condition

37 $$\begin{aligned} \lambda t\sqrt{N}=\frac{\pi }{2} (1+2m), \quad m=0,1,2,... \end{aligned}$$

and substitute Eq. (37) into Eq. (36) to obtain the relation

38 $$\begin{aligned} \cos \left( \pi (1+2m)\sqrt{1-\frac{1}{2N}}\right) =1,\quad m=2N. \end{aligned}$$

The condition is fulfilled for large *N*:

39 $$\begin{aligned} \cos \left( \pi (1+4N)\sqrt{1-\frac{1}{2N}}\right) \approx 1-\frac{1}{2}\left( \frac{1\pi }{4N}\right) ^2\xrightarrow [N\rightarrow \infty ]{}1 \end{aligned}$$

From Eq. (37), the product of the parameters $\lambda$ and *t* is given by

40 $$\begin{aligned} \lambda t=\frac{\pi }{2}\frac{1+4N}{\sqrt{N}}. \end{aligned}$$

The parameter $\lambda$ which is the interaction strength of an atom with the mode in the strong coupling regime can be estimated as a few tens of gigahertz. In papers^62–66^ the experimental results were obtained and the theory of interaction of a microresonator mode with atoms located inside the resonator is proposed. Assuming that $\lambda =2\pi \times 10$ GHz and $N=10000$ the estimate for *t* is $t\approx 10^{-8}$ s. Thus, in the limiting case, where $N\rightarrow \infty$ and Eq. (36) is satisfied, the Kraus operators from Eqs. (33)–(35) can be expressed as follows:

41 $$\begin{aligned} K_0=|0\rangle \langle 0|+|2\rangle \langle 2|,\quad K_1=|0\rangle \langle 1|,\quad K_2=0\cdot |0\rangle \langle 2|. \end{aligned}$$

In other words this means that probability of finding two exited atoms vanishes. Filter operators can now be constructed from the operators that measure the photon numbers in each spatial mode on each of the four channels. We will label the operators with an additional index $k=1,2,3,4$, which denotes the number of the channel. So, in what follows we will use the notation $K^{(k)}_0,K^{(k)}_1, K^{(k)}_2$. The condition $K^{(k)}_2=0$ denotes that under the chosen interaction conditions, two excited atoms are not observed in the channel. Operators $K^{(k)}_0$ and $K^{(k)}_1$ act as follows:

42 $$\begin{aligned}&K^{(k)}_1 (\alpha |0\rangle +\beta |1\rangle +\gamma |2\rangle )= \beta |0\rangle , \end{aligned}$$

43 $$\begin{aligned}&K^{(k)}_0 (\alpha |0\rangle +\beta |1\rangle +\gamma |2\rangle )= \alpha |0\rangle +\gamma |2\rangle . \end{aligned}$$

Thus the filtration operators for the introduced measurement can be described as follows:

44 $$\begin{aligned} F_1= & {} \frac{1}{\sqrt{2}} \left( K_0^{(1)}K_0^{(2)}K^{(3)}_1 K_1^{(4)}-K^{(1)}_1 K_1^{(2)}K_0^{(3)}K_0^{(4)}\right) , \end{aligned}$$

45 $$\begin{aligned} F_2= & {} \frac{1}{\sqrt{2}} \left( K^{(1)}_1 K_1^{(4)}K_0^{(2)}K_0^{(3)}-K^{(2)}_1 K_1^{(3)}K_0^{(1)}K_0^{(4)}\right) , \end{aligned}$$

46 $$\begin{aligned} F_3= & {} K^{(1)}_0 K_0^{(2)}K_0^{(3)}K_0^{(4)} \end{aligned}$$

Clearly, the operator $F_1$ acts on the state $|D_1\rangle$ as follows:

47 $$\begin{aligned} F_1|D_1\rangle =|0\rangle _1|0\rangle _2|0\rangle _3|0\rangle _4 \end{aligned}$$

and the probability of the result is given by

48 $$\begin{aligned} P_1=\langle D_1|F_1^\dagger F_1|D_1\rangle =1. \end{aligned}$$

As a result, this measurement is unambiguous and, in this case, other events cannot be observed.

#### Unitary operation

As is shown in Fig. 2, the second step of the protocol is executed depending on the value of the classical bit. When the bit equals zero, no further unitary operations are required. In the opposite case where the value of the bit is unity, an additional unitary operation should be applied.

In our case, this unitary operation can be realized using two 50:50 beam splitters connecting the channels 1 and 2, 3 and 4, respectively.

Relation describing the state transformation under the action of filtration and an appropriate unitary operation is now given by

49 $$\begin{aligned}&|D_3\rangle \xrightarrow {U^\Phi } \frac{1}{2\sqrt{2}}\left( a_{3}^{\dagger 2}- a_{1}^{\dagger 2} + a_{4}^{\dagger 2} -a_{2}^{\dagger 2}\right) |0\rangle , \end{aligned}$$

50 $$\begin{aligned}&|D_4\rangle \xrightarrow {U^\Phi } \frac{1}{\sqrt{2}}\left( a_{3}^\dagger a_{4}^\dagger - a_{1}^\dagger a_{2}^\dagger \right) |0\rangle . \end{aligned}$$

After the unitary rotation one can apply again the allocation of single photon subspace and unambiguously discriminate between the states $|B_3\rangle$ and $|B_4\rangle$. Thus, according to the proposed two-step Bell state measurement scheme, one can discriminate all four of them unambiguously.

## Discussion

In this paper we have proposed the filtration mapping realizing complete Bell state analyzer of photonic qubits. The key distinctive feature of our approach is that it avoids using either hyperentanglement or the representation of the Bell states as concatenated Greenber–Horne–Zeilinger (C-GHZ) state to provide the discrimination. In addition, the approach is flexible so that the method can be implemented in different ways. We have described the two different realizations of such filtration mapping for complete Bell state measurement. In non-demolition measurement, we separate two pairs of the states from each other as the filtration process utilizing of the cubic (Kerr) nonlinearity and the probe mode. In semi-demolition measurement, the two states are unambiguously discriminated and thus destructed, whereas the other states passes the filter without modification. It can be organized as follows. The measurement destroys the single photon subspace in every mode and preserves the superposition of zero and two photons. It can be realized with discrete photodetection based on microresonator with atoms^59^. It can be considered as quadratic nonlinearity just as any particular kind of photodetection.

We conclude with the remark that previously, even in principle, it was impossible to provide the deterministic discrimination of polarization qubits without their special transformation or utilization of auxillary degrees of freedom to other quantum states due to the fact of that photons are bosonic particles and thus “bunching together”. The result offers unique opportunity to perform deterministic realizations of various quantum protocols, e.g. quantum teleportation, dense coding, quantum repeaters, measurement-device-independent QKD.
